# Role of Taste Receptors as Sentinels of Innate Immunity in the Upper Airway

**DOI:** 10.1155/2018/9541987

**Published:** 2018-10-01

**Authors:** Neil N. Patel, Alan D. Workman, Noam A. Cohen

**Affiliations:** ^1^Department of Otorhinolaryngology-Head and Neck Surgery, Division of Rhinology, University of Pennsylvania, Perelman School of Medicine, Philadelphia, PA, USA; ^2^Corporal Michael J. Crescenz Veterans Administration Medical Center, Philadelphia, PA, USA; ^3^Monell Chemical Senses Center, Philadelphia, PA, USA

## Abstract

Evidence is emerging that shows taste receptors serve functions outside of taste sensation of the tongue. Taste receptors have been found in tissue across the human body, including the gastrointestinal tract, bladder, brain, and airway. These extraoral taste receptors appear to be important in modulating the innate immune response through detection of pathogens. This review discusses taste receptor signaling, focusing on the G-protein–coupled receptors that detect bitter and sweet compounds in the upper airway epithelium. Emphasis is given to recent studies which link the physiology of sinonasal taste receptors to clinical manifestation of upper airway disease.

## 1. Introduction

Recent evidence suggests that the role of taste receptors extends beyond mere taste sensation that drives our preferences for foods. In fact, taste receptors have been found in extra-oral tissues throughout the body, including the airway, gastrointestinal tract, pancreas, bladder, and ventricles of the brain [1–4]. These extra-oral taste receptors have demonstrated importance in modulating local innate immunity [1–4]. In this expanded functional paradigm, taste receptors serve as the sentinels of pathogenic detection and, therefore, are thought to mediate the balance between commensalism and pathogenicity [5, 6].

This review will focus on the function of bitter and sweet taste receptors in the human airway and will highlight studies demonstrating how taste receptors trigger innate immune responses. Emphasis will also be given to recent literature implicating bitter and sweet taste receptor function in the clinical manifestation of human upper airway inflammation. We will conclude with future directions that are emerging around the diagnostic and therapeutic potential of taste receptors.

## 2. Bitter and Sweat Taste Receptor Signaling

Bitter and sweet taste receptors, unlike ion-stimulated taste receptors for saltiness and sour sensation, are G-protein Coupled Receptors (GPCRs) [7, 8]. In the human tongue, perception of sweet molecules is mediated through a family of GPCRs, Taste Receptor Family 1 (T1R), within which there are two isoforms called T1R2 and T1R3 [9–12]. T1R2/3 are stimulated by sugars, including glucose, sucrose, and fructose [9, 13, 14]. On the other hand, bitter perception is detected by approximately 25 different isoforms of Taste Receptor Family 2 (T2R), the collective profile of which has been referred to as the “bitterome” [15, 16]. This larger and more heterogenous family of T2Rs responds to an assortment of bitter compounds [17], including sesquiterpene lactones, strychnine, and denatonium [18]. A single bitter agonist can stimulate multiple T2R isoforms, and a single receptor isoform can respond to multiple agonists, thereby creating a redundant pattern of encoding [19]. Furthermore, among the 25 T2Rs, the collective receptive range of 19 receptors accounts for approximately 80% of the established bitter library; the handful of remaining “orphan” receptors have ligands that are yet to be identified [19].

The mechanisms involved in taste receptor activation are relatively conserved and follow similar pathways in the tongue and extra-oral tissues. When a ligand binds to a taste GPCR, associated G-proteins initiate a downstream cascade. Chief among taste-associated G-proteins is gustducin, which comprises G*α* and G*βγ* subunits. In addition, inhibitory alpha subunits called G*α*i are capable of attenuating the signal transduction cascade. While cascade activation via *α* gustducin is well described, characterization of inhibitory G*α*i signaling has not been as well studied. Nevertheless, murine models in which gustducin is knocked out still demonstrate residual taste function, which has led to the belief that other G-proteins, namely, G*α*14, G*α*15, G*α*q, G*α*s, G*α*i-2, and G*α*i-3, may also play a role in taste signal transduction [20–22].

Stimulation of GPCRs via G*α* leads to downstream activation of Phospholipase C isoform *β*2 (PLC*β*2), which in turn produces downstream inositol 1,4,5-trisphosphate (IP3). Activation of the IP3 receptor on the endoplasmic reticulum releases calcium into the cytosolic compartment [23]. Simultaneously, there is also an activation of phosphodiesterases (PDEs) that attenuate cyclic adenosine monophosphate (cAMP) levels, in effect decreasing protein kinase A (PKA) activity. Since PKA is an inhibitor of the IP3 receptor isoform, release of IP3 receptor inhibition allows for further calcium efflux from the endoplasmic reticulum [24]. Cytosolic calcium activates the nonselective cation channel, transient receptor potential cation channel subfamily M member (TRPM5), causing plasma membrane depolarization, which activates voltage-gated sodium (Na^+^) channels, resulting in action potential propagation that ultimately causes ATP release through calcium homeostasis modulator 1 (CALHM1), a large pore channel [9, 24–26]. In the tongue, ATP release activates receptors on taste cells and sensory fibers that transmit sensations to the central nervous system [9, 26]. As it pertains to airway taste receptor signaling, the upstream activation of GPCRs results in a calcium wave transmitted through airway epithelial gap junctions. This calcium wave is critical for driving tissue-level innate immune defenses, which we will discuss further below.

## 3. Taste Receptors in Respiratory Innate Immunity

### 3.1. Overview of Airway Innate Immunity

The sinonasal tract (i.e., nose and paranasal sinuses) represents one of the body's first points of contact with the external environment, which is fraught with respiratory pathogens. Despite the continuous onslaught, the upper airway innate immune defense is capable of preserving the sterility found in the lower airway and lung parenchyma [27]. To accomplish this, the upper airway features multiple components aimed at combating pathogens.

**Figure 1:**
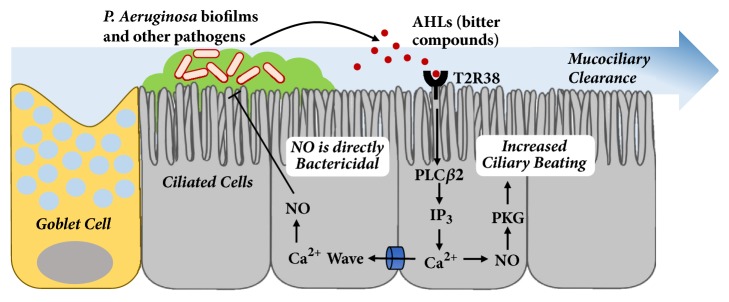
Bitter taste receptor, T2R38, is expressed on ciliated cell and mediates innate immune response to bitter compounds, such as AHLs secreted by* P. aeruginosa.* Innate immune defences include nitric oxide (NO) production as well as increased ciliary beating.

The main arsenal of upper airway innate immunity is mucociliary clearance (Figure 1). Mucus is a sticky gel comprised of cross-linked glycosylated mucin macromolecules produced by airway goblet cells [28]. Inspired pathogens and particles are trapped in mucus. Cilia on respiratory epithelial cells beat in a spatially and temporally organized pattern to clear the debris and pathogen-laden mucus out of the paranasal sinuses and nasal cavity [28]. This system is complemented with the production of nitric oxide (NO) and other antimicrobial agents that are produced by respiratory epithelial cells. Lastly, the respiratory epithelial cells have been shown to secrete their own set of cytokines and chemokines, such as interleukin 25 (IL-25), thymic stromal lymphopoietin (TSLP), and interleukin 33 (IL-33), which trigger downstream immune cascades [29–31]. Studies have shown that taste receptors are at the front-line when it comes to detecting pathogens and modulating these innate immune defense mechanisms.

### 3.2. Taste Receptors as Detectors of Pathogens

Bitter taste receptors are expressed in upper respiratory epithelium and respond to bitter molecules released by pathogens in the mucosal environment [32–34]. A prime example of bitter taste receptor detection of pathogens is demonstrated by lactones, a class of bitter compounds, which includes acyl-homoserine lactones (AHLs) that are produced by many gram-negative bacteria [35, 36]. These lactones serve as biofilm “quorum-sensing molecules;” bacteria will initiate biofilm formation when a high enough concentration of AHLs is reached in a localized area. Biofilms can provide protection for bacteria from host innate immune defenses as well as antibiotics [37]. It is hypothesized that bitter taste receptors “eavesdrop” on these bacterial communications, effectively detecting AHLs before a sufficient concentration is reached for biofilm formation [5]. The bitter taste receptors themselves elicit innate immune responses that can eradicate bacteria before pathogenic levels are achieved.

Once they are activated, bitter taste receptors engage innate immune defenses to fight off pathogens. In particular, bitter receptors expressed on ciliated cells have been found to be stimulated by bacterial compounds and cause downstream release of nitric oxide (NO), as shown in Figure 1 [38, 39]. NO diffuses quickly into bacteria, where it destroys intracellular components [32, 40]. Some bacteria, such as* P. aeruginosa*, are highly sensitive to NO, while others are more resistant [41]. In addition to this antimicrobial activity, NO also activates protein kinase G (PKG) and guanylyl cyclase to directly speed up ciliary beat frequency (CBF), increasing mucociliary clearance (Figure 1) [42]. Rapid ciliary beating can clear bacteria and mucus out of the paranasal sinuses and nasal cavity into the throat, where they can be expectorated or swallowed. Additionally, ciliary beating helps disperse antimicrobial products, such as lactoferrin, lysozyme, and defensins, across the surface of airway mucosa [28]. These antimicrobial compounds act in concert with NO and other reactive oxygen species to create a potent antipathogenic response [40].

As shown in Figure 1, T2R38 is a bitter taste receptor located on ciliated cells in humans, and it responds to at least three AHLs produced by* P. aeruginosa*: N-butyrl-L-homoserine lactone, N-hexanoyl-L-homoserine lactone, and N-3-oxo-dodecanoyl-L-homoserine lactone [32]. In addition to its response to bacterial compounds, T2R38 reacts in a similar fashion to the bitter compounds, phenylthiocarbamide (PTC), and propylthiouracil (PROP) [43]. In response to PTC stimulation, sinonasal epithelial cells expressing a functional T2R38 receptor demonstrate a substantial increase in NO production (Figure 1). Interestingly, the taste G-protein gustducin does not appear to be involved [32].

The recognition of pathogens by taste receptors and nearly immediate release of antipathogenic compounds highlights a unique physiologic characteristic. It is well established that Toll-like receptors (TLRs) respond to pathogen-associated molecular patterns (PAMPs), which include foreign cellular components. However, TLR signaling is gradual, taking up to 12 hours to exert an immune response through changes in expression of genes that play a role in innate immunity [44]. Conversely, bitter taste receptors can detect bacterial products, such as AHLs, and elicit downstream increases in immune defense in a much more expedient fashion—on the order of seconds to minutes.

### 3.3. Solitary Chemosensory Cells

Ciliated epithelial cells are not the only cells to express bitter taste receptors in the airway. Over a decade ago, a class of cells that is sparsely scattered in rodent respiratory epithelium was shown to be immunoreactive with alpha-gustducin, a component of taste signaling [45]. Analogous “taster” cells are also found in the human upper airway [30, 33]. These cells were named “solitary chemosensory cells” (SCCs), and they share many similarities with cells found in the taste buds of the tongue [34]. In rodents and humans alike, approximately one out of every hundred epithelial cells in the sinonasal cavity is an SCC, and despite their rarity, studies have implicated their function in innate immune responses [46].

**Figure 2:**
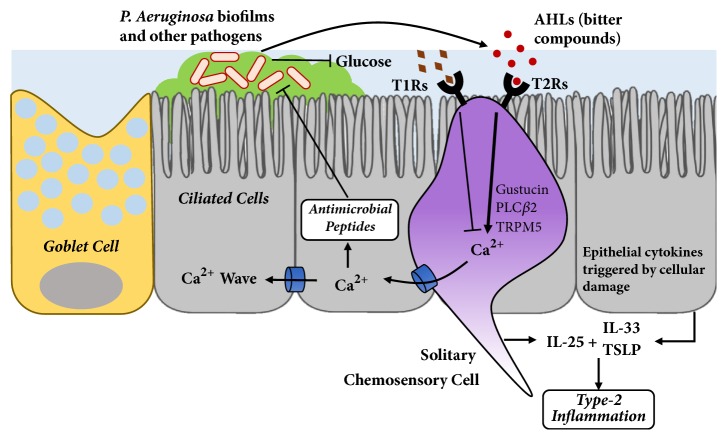
Solitary chemosensory cells (SCCs) express counteracting sweet receptors (T1Rs) and bitter receptors (T2Rs) that can cause a calcium wave leading to release of antimicrobial peptides. Solitary chemosensory cells have separately also been found to release innate cytokine, IL-25, and epithelial cells produce IL-33 and TSLP in the setting of cellular damage.

In murine models, SCCs express sweet and bitter taste receptors [33, 47], and they are capable of responding to AHLs and other bitter agonists (Figure 2) [5, 48, 49]. While SCCs show intracellular calcium responses in the presence of AHLs [50], they differ from ciliated cells in that they do not activate downstream NO production. Instead, when mouse sinonasal SCCs are stimulated with AHLs or the bitter agonist denatonium, the calcium response results in acetylcholine (ACh) release that stimulates trigeminal nerve peptidergic nociceptors, with downstream effects of breath holding and inflammatory mediator release [5, 46, 48]. The inflammatory response is intuitively antimicrobial, while the breath holding response may also represent an adaptive reflex to limit toxin or organism aspiration in the host. Calcitonin gene related peptide (CGRP), substance P, and vasoactive intestinal peptide (VIP) are known substances released in this inflammatory cascade [51].

SCCs have been identified in human upper airway tissue as well [33, 52], along with additional physiological function beyond what has been elucidated in the rodent system. T1R1, T1R2, T2R4, T2R10, and T2R47 are all expressed on SCCs in the human nasal cavity [52, 53]. Denatonium, a bitter compound that shows activity in the murine SCC, also stimulates a Ca^2+^ response in human SCCs that spreads to neighboring cells via gap junctions (Figure 2) [53]. Just as in the NO response seen in ciliated cells, the calcium signaling requires many known components of traditional taste signaling, including gustducin, PLC*β*2, the IP_3_ receptor, and TRPM5 [53]. Spread of calcium through gap junctions causes immediate release of antimicrobial peptides (AMPs) from the adjacent ciliated cells [33]. These AMPs include beta defensin 1 (BD1) and beta defensin 2 (BD2), which have potent antimicrobial effects on gram-positive and gram-negative organisms, including methicillin-resistant* S. aureus *and* P. aeruginosa* [51].

Interestingly, denatonium-induced calcium waves initiated by SCCs are inhibited in a dose-dependent fashion by sugars, such as glucose and sucrose (Figure 2) [53]. This inhibition has also been observed with nonmetabolizable artificial sweetener, sucralose, a potent T1R2/3 agonist [12, 13, 54]. The glucose or sucralose inhibition appears to be reversed by T1R2/3 antagonists lactisole [10, 55] and amiloride [56], but not by inhibitors of glucose transporters such as phloretin and phlorizin [53]. Taken together, these studies demonstrate that bitter and sweet signals have opposing effects on innate immunity, as illustrated in Figure 2. Linking these findings to the overall paradigm, we posit that as pathogens consume sugars as an energy source, increased T1R2/3 signaling augments the calcium wave that is also directly driven by T2R detection of bitter molecules secreted by the pathogens, thereby eliciting an even more robust innate immune response. In support of this, patients with diabetes and prediabetes have been found to have elevated glucose levels in nasal secretions [57] and are more likely to have airway infections [58].

More recently, human SCCs have also been found to be the source of epithelial-derived cytokine, IL-25 [30, 31]. Unlike hematopoietic cytokines, epithelial-derived cytokines, namely, IL-25, TSLP, and IL-33, are released when airway epithelium is exposed to allergens, fungi, and viral antigens (Figure 2) [59]. These cytokines act on resident group-2 innate lymphoid cells which trigger an eosinophilic type-2 inflammatory cascade [60]. While evidence demonstrates that SCCs expand and increase production of IL-25 in inflamed sinonasal tissue [30], it remains unknown if bitter agonists such as AHLs and denatonium are capable of triggering IL-25 release. Further studies are warranted to elucidate how bitter agonists might play a role in triggering epithelial-derived cytokines.

## 4. Genetics of Taste Receptors and Clinical Correlates

While genetic variation in T2Rs giving rise to differential tasting ability has been well studied, the link between genetic polymorphisms of T2Rs and pathogenic susceptibility is just now coming to light. The theory is that dysfunctional bitter taste receptors will not allow patients to mount a robust immune response against pathogenic infection, thereby leaving them more vulnerable to sinonasal disease.

It is well established that the genetic locus for T2R38,* TAS2R38*, has common polymorphisms that can render the receptor nonfunctional. Individuals with a proline-alanine-valine (PAV) amino acid sequence at a key portion of the taste receptor are able to respond to T2R38 agonists, while individuals with an alanine-valine-isoleucine (AVI) sequence at this same locus possess a nonfunctional receptor variant [19]. Cells isolated from individuals with an AVI/AVI genotype show highly attenuated NO production in response to AHLs, PTC, or PROP stimulation, compared to cells isolated from individuals with a PAV/PAV genotype [32]. Downstream reductions in mucociliary clearance and bacterial killing are correspondingly observed [32]. As would be expected, AVI/AVI individuals also do not taste PTC or PROP when presented with an oral taste test challenge.

This reduction in responsiveness observed in AVI-expressing individuals has clinical consequences. Several studies in the past five years have highlighted a potential relevance of T2R38 in chronic rhinosinusitis (CRS). Individuals who express the fully functional, PAV/PAV, genotype are less likely to require surgical intervention for CRS symptoms than patients with an AVI/AVI genotype [61–63]. Additionally, levels of gram-negative infection are lower in PAV/PAV patients [55, 61, 62, 64–66], confirming that the NO-dependent response of T2R38 acts as a critical defense for this class of bacteria. A hallmark of chronic rhinosinusitis is mucociliary stasis, in which bacteria are inadequately cleared. At pathogenic levels of proliferation, bacterial toxins can be destructive to cells and cilia, perpetuating the process of impaired mucociliary function [67]. It is known that sinonasal explants from patients with CRS have an attenuated response to a variety of compounds (bitter and non-bitter) that stimulate ciliary beating in control tissue. Other studies, while part of an inconclusive set of literature, have shown differences in NO levels in patients with airway diseases [68]. Without the action of NO to kill bacteria and increase ciliary beating in response to AHLs, it appears that the nonfunctional T2R38 polymorphism has a phenotypic effect on upper airway disease [32].

Other bitter taste receptors on ciliated cells, such as T2R4 and T2R14 [69], respond to different bitter agonists, such as quinine hydrochloride. Quinine is an alkaloid derivative that is isolated from the cinchona tree and is found in several medicinal and commercial products [70]. Recent work shows that quinine stimulates a rapid T2R-dependent NO response from ciliated cells in the airway [71]. While quinine is a more promiscuous bitter taste receptor agonist than PTC or PROP, there are common genetic variants in bitter taste receptor genes on chromosome 12 that strongly contribute to perception of quinine taste intensity [72]. Quinine taste sensitivity also has been selected independently in some world populations, especially at low concentrations of quinine [73]. Concentrations of bitter microbial products in the airway are also at low concentrations [32], and these differences in taste perception of dilute quinine solutions may be reflective of varying responses of these bitter taste receptors in both the airway and on the tongue. Allele expression studies have shown that patients with CRS differ from control patients at several genetic loci for taste receptors, including TAS2R14 and TAS2R49 [64]. Indeed, further genetic studies elucidating the relationship between the human “bitterome” and clinical manifestations of upper airway disease are warranted.

## 5. Future Diagnostic and Therapeutic Potential of Taste Receptors

Given that genetic variation in bitter taste receptors appears to be correlated with disease status and severity, testing phenotypic function of taste receptors may have clinical utility. Using subjective taste intensity scoring schema, patients with chronic rhinosinusitis reported lower intensity of taste of bitter compounds compared to matched healthy controls, but these same, chronic rhinosinusitis patients reported higher intensity of sucrose, a T1R agonist, compared to their healthy counterparts [71, 74]. These subjective taste differences also appear to be reflected at the physiologic level; experiments have shown an inverse association between* in vitro* biofilm formation and PTC taste intensity ratings [75].

**Figure 3:**
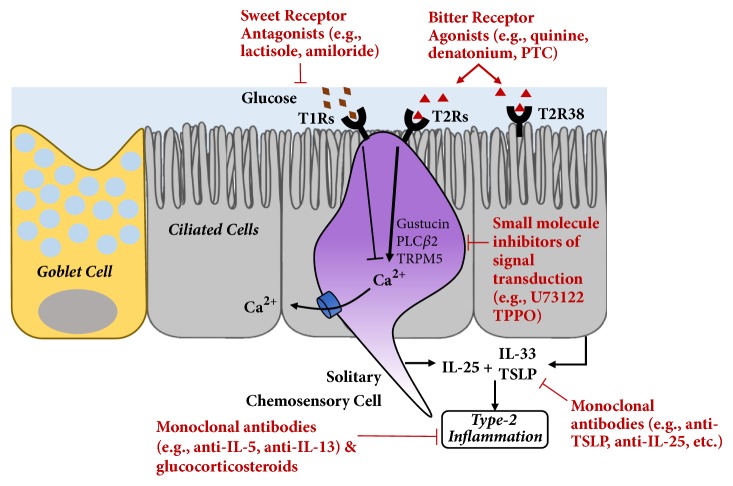
Potential targets for therapeutic intervention in the taste receptor pathway. Inhibitors of sweet receptors and agonists of bitter taste receptors may potentiate innate immune responses from ciliated cells and SCCs. In addition, small molecular inhibitors are known to block signal transduction but have only been used experimentally. Inhibitory monoclonal antibodies, such as anti-TSLP as well as anti-IL-5 and IL-13, have also recently entered the market or are undergoing clinical investigation (U73122 inhibits Phospholipase C; TPPO = triphenylphosphine oxide inhibits TRMP5).

Oral taste tests are inexpensive to produce and administer, and the ability to assess variations in airway taste receptor functionality could help predict impaired innate immunity or predisposition to respiratory disease. Bitter taste testing with specific agonists, such as PTC, could potentially be used to stratify surgical candidates or identify individuals who should receive more aggressive management. Beyond the diagnostic realm, bitter taste receptor agonists may have therapeutic potential (Figure 3) in harnessing potent innate immune defenses as an alternative to more conventional treatments, such as antibiotics.

## 6. Conclusion

Extra-oral taste receptors in the nose and paranasal sinuses serve to detect pathogens and modulate innate immune responses. Bitter taste receptors can detect bacterial by-products, while sweet receptors are thought to release inhibition on the taste cascade when pathogens deplete glucose on the apical microenvironment. The resulting downstream calcium wave leads to rapid release of antipathogenic nitric oxide and antimicrobial peptides. Human genetic polymorphisms in bitter taste receptors correlate with sinonasal disease and can be evaluated through oral taste testing. Taste receptors represent a new frontier when it comes to their diagnostic and therapeutic potential.
